# Genetic variation and genetic complexity of nodule occupancy in soybean inoculated with USDA110 and USDA123 rhizobium strains

**DOI:** 10.1186/s12864-023-09627-4

**Published:** 2023-09-04

**Authors:** Susan Araya, Patrick Elia, Charles V. Quigley, Qijian Song

**Affiliations:** Soybean Genomics and Improvement Laboratory, USDA-ARS, Beltsville, MD 20705 USA

**Keywords:** Soybean, rhizobium, USDA110, USDA123, GWAS, Nodulation restriction

## Abstract

**Background:**

Symbiotic nitrogen fixation differs among *Bradyrhizobium japonicum* strains. Soybean inoculated with USDA123 has a lower yield than strains known to have high nitrogen fixation efficiency, such as USDA110. In the main soybean-producing area in the Midwest of the United States, USDA123 has a high nodule incidence in field-grown soybean and is competitive but inefficient in nitrogen fixation. In this study, a high-throughput system was developed to characterize nodule number among 1,321 *Glycine max* and 69 *Glycine soja* accessions single inoculated with USDA110 and USDA123.

**Results:**

Seventy-three *G. max* accessions with significantly different nodule number of USDA110 and USDA123 were identified. After double inoculating 35 of the 73 accessions, it was observed that PI189939, PI317335, PI324187B, PI548461, PI562373, and PI628961 were occupied by USDA110 and double-strain nodules but not by USDA123 nodules alone. PI567624 was only occupied by USDA110 nodules, and PI507429 restricted all strains. Analysis showed that 35 loci were associated with nodule number in *G. max* when inoculated with strain USDA110 and 35 loci with USDA123. Twenty-three loci were identified in *G. soja* when inoculated with strain USDA110 and 34 with USDA123. Only four loci were common across two treatments, and each locus could only explain 0.8 to 1.5% of phenotypic variation.

**Conclusions:**

High-throughput phenotyping systems to characterize nodule number and occupancy were developed, and soybean germplasm restricting rhizobium strain USDA123 but preferring USDA110 was identified. The larger number of minor effects and a small few common loci controlling the nodule number indicated trait genetic complexity and strain-dependent nodulation restriction. The information from the present study will add to the development of cultivars that limit USDA123, thereby increasing nitrogen fixation efficiency and productivity.

**Supplementary Information:**

The online version contains supplementary material available at 10.1186/s12864-023-09627-4.

## Background

Soybean [*Glycine max* (L.) Merr.] is one of the most economically important crops in the world due to its rich protein and oil content, about 42 and 20%, respectively [1, 2]. Protein content in soybean requires a great demand for nitrogen which is supplied by biological symbiotic nitrogen fixation with the bacteria bradyrhizobia that fix atmospheric nitrogen and by the residual soil nitrogen pool [3, 4]. It is estimated that the nitrogen in soybean seeds derived from symbiotic fixation is 25–50% in soils with a large amount of mineral nitrogen and 80–94% in soils with low organic matter and nitrogen [5, 6].

Variability in symbiotic nitrogen fixation among different strains of *B. japonicum* has been observed. For instance, several authors have reported that the seed yield of soybeans grown in rhizobia-free soil inoculated with *B. japonicum* USDA123 was lower than in plants inoculated with strains known to have high nitrogen fixation efficiency, such as USDA110 [7–9]. In the Midwestern of the United States, the primary soybean production region, the incidence of *B. japonicum* strain USDA123 in field-grown soybean nodules is high [10–15], and the strain is highly competitive for nodule sites [11, 16, 17].

Since the early days, many attempts have been made to increase soybean nitrogen fixation efficiency through the use of superior inoculant strains like USDA110 [7, 18], improving the inoculation method [13, 19, 20], creation of mutants or genetically modified rhizobium strains with an increased ability to fix nitrogen [21–23], and selection of genotypes [16, 24–27]. Among them, identifying soybean accessions that restrict nodulation with indigenous serocluster 123 strain and developing soybean cultivars that can limit low-efficiency nitrogen-fixation strains are economical and effective ways to increase soybean symbiotic nitrogen fixation and, eventually, productivity.

The Soybean Germplasm Collection of the United States Department of Agriculture includes 18,480 cultivated and 1,168 wild soybean accessions introduced from 84 countries or developed in the United States [28]. This collection was genotyped with the SoySNP50K BeadChip assay containing 52,509 single nucleotide polymorphisms (SNPs) at the Soybean Genomics and Improvement Laboratory, Beltsville, Maryland, USA. The SoySNP50K dataset greatly facilitated the elimination of redundant accessions and the selection of a diverse set of germplasm from different countries for discovering individuals with restriction of strain USDA123 nodulation or individuals with preference to nodulate with efficient nitrogen-fixation strains. Thus, it is possible to combine nodulation restriction of the indigenous serocluster 123 strain and nodulation favoring one or more efficient strains into one genotype. The germplasm and the genotypic dataset are also valuable resources for understanding the complexity of the traits by examining the genes or genomic regions controlling nodulation restriction and preferences. Several genes restricting nodulation with other Bradyrhizobia strains have been published, e.g., *Rj*2 restricts nodulation of *B. japonicum* strain USDA122 [29, 30], the *Rj*_3_ locus restricts nodulation of *B. elkanii* strain USDA33, the *Rj*_*4*_ locus restricts *B. elkanii* strain USDA61 [31, 32], and *Rfg1* restricts nodulation of *Ensifer*/*Sinorhizobium fredii* strain USDA257 [33]. In addition, soybean carrying the homozygotic recessive *rj*_*1*_, *rj*_*5*_, and *rj*_*6*_ alleles restrict all rhizobium strains [34]. Nevertheless, genetic control of restricted nodulation with USDA123 or preference with USDA110 has not been reported.

Therefore, the objectives of the present study were to determine the genetic variation of nodule number (NN) among sets of diverse cultivated and wild soybean accessions after single inoculation with *B. japonicum* strains USDA110 and USDA123, to identify new germplasm that may restrict nodulation of USDA123 but prefer USDA110 strain by characterizing nodule occupancy in soybean double inoculated with USDA110 and USDA123, and to determine genetic complexity of the NN trait in soybean accessions.

## Results

### Genetic variation of nodule number among soybean accessions single inoculated with USDA110 and USDA123

The NN was counted on 1,321 accessions of *G. max* after single inoculation with *B. japonicum* strains USDA110 and USDA123, respectively (Table S1). Two of the *G. max* accessions did not nodulate with any of the two strains tested. They were grown in field soil and confirmed no nodules on their roots. The non-nodulated accessions tested were PI548193 (T201), a well-known accession with genotype *rj1* non-nodulating, and PI507429 (Tousan 89) introduced to the U.S. from Japan in 1987 (www.ars-grin.gov) which has not been previously reported with restricted nodulation.

The average NN of the 1,321 *G. max* accessions was 35.3 nodules per plant and ranged from 0 to 75.3 when inoculated with USDA110, and the average was 33.8 nodules per plant, ranging from 0 to 75 when inoculated with USDA123. The average number of nodules of *G. soja* was 12.9 and 13.8 per plant, ranging from 5.3 to 23.5 and 2.3 to 27.8 when inoculated with USDA110 and USDA123, respectively. The NN in both cultivated and wild soybean were normally distributed with Skewness between − 2 to + 2 and Kurtosis between − 7 to + 7 (Table 1), which were the thresholds for normal distribution. The heritability in *G. max* single inoculated with USDA110 and USDA123 were 0.70 and 0.68, respectively, and similar results were found in *G. soja* when inoculated with USDA110 (0.6) and USDA123 (0.74). The phenotypic variation of NN in *G. max* and *G. soja* was mainly attributed to genetic effects, and the trait was less affected by the environment, i.e., relatively high consistency of NN among replicates in the experiment.

**Table 1:** Distribution of nodule number (NN) per plant in the diverse sets of *G. max* and *G. soja* accessions single inoculated with *B. japonicum* strain USDA110 and USDA123

Treatment	Species	Number of accessions	Mean + SD	Range	Kurtosis	Skewness
NNmax110	*G. max*	1,321	35.3 ± 9.5	0.0–75.3	1.29	0.66
NNmax123	*G. max*	1,321	33.8 ± 9.3	0.0–75.0	0.82	0.70
NNsoja110	*G. soja*	69	12.9 ± 3.9	5.3–23.5	0.34	0.57
NNsoja123	*G. soja*	69	13.8 ± 8.2	2.3–27.8	1.13	0.86

**Fig. 1:**
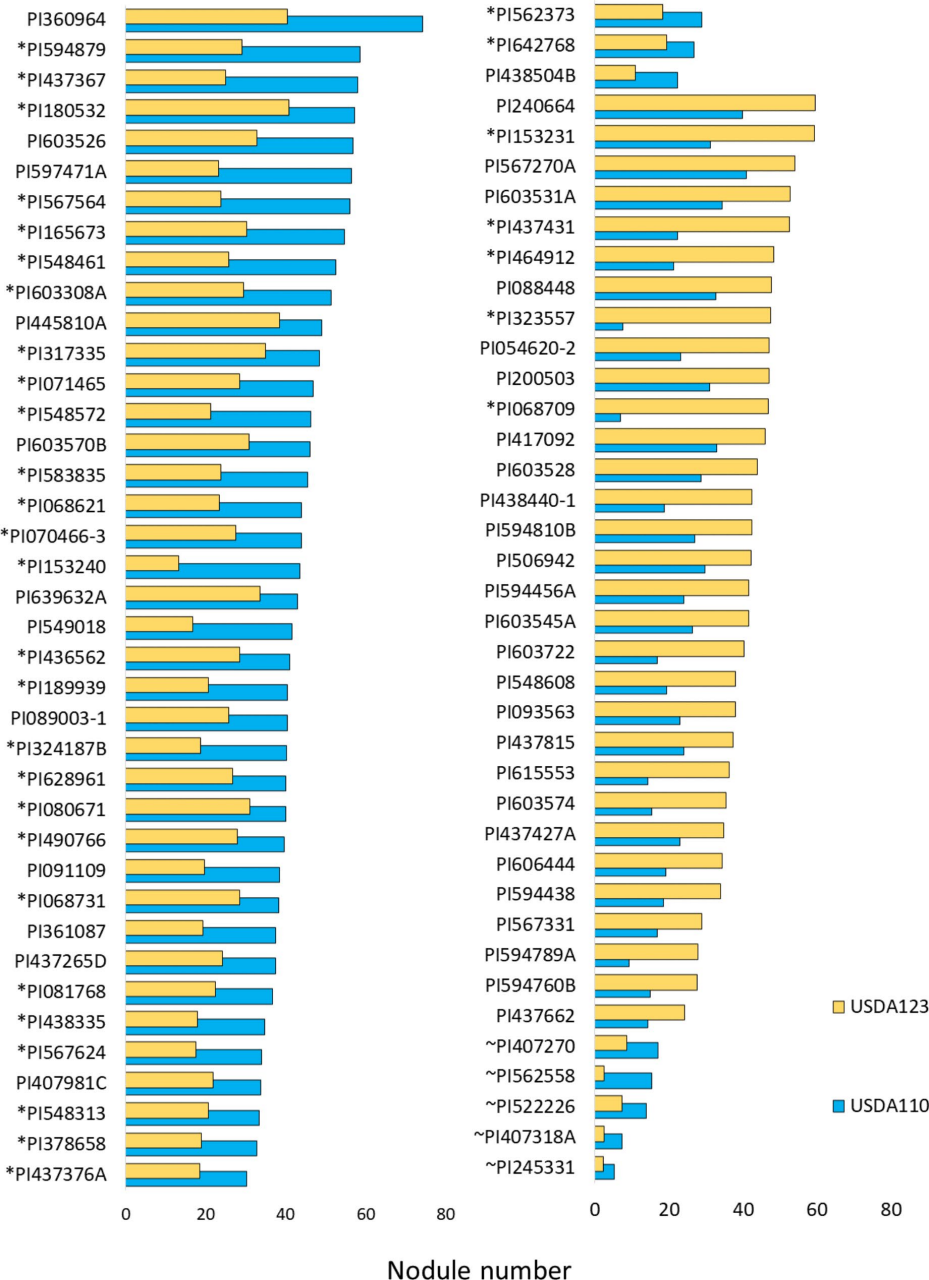
Nodule number of 73 *G. max* and five *G. soja* (prefixed with ~) accessions with a significant difference in nodule number after inoculation with *Bradyrhizobium japonicum* strains USDA110 and USDA123 (P < 0.05), respectively. The accessions prefixed with an asterisk (*) were chosen to be inoculated with mixed USDA110 and USDA123 inoculum for the subsequent competition test

A t-test (*P* < 0.05) was performed to determine the statistical difference in NN of USDA110 vs. USDA123 in 1,319 cultivated, and 69 wild soybean accessions single inoculated with USDA110 and USDA123, respectively. The non-nodulated accessions were not included in this analysis. Forty-two cultivated soybean accessions showed significantly higher NN of USDA110 than USDA123, and 31 had a lower NN of USDA110 than USDA123 (Table S2). Among these, the top ten accessions of cultivated soybean with a high NN of USDA110 and a low NN of USDA123 in single inoculation were PI360964, PI594879, PI437367, PI180532, PI603526, PI597471A, PI567564, PI165673, PI548461, and PI603308A. Conversely, the top 10 accessions with low NN of USDA110 but high NN of USDA123 were PI240664, PI153231, PI567270A, PI603531A, PI437431, PI464912, PI088448, PI323557, PI054620-2 and PI200503 (Fig. 1).

Besides, five wild soybean accessions, PI562558, PI407270, PI522226, PI407318A, and PI245331, had significantly higher NN when nodulated with USDA110 than USDA123, but no accession with more USDA123 than USDA110 nodules was found.

### Nodule occupancy in soybean double inoculated with USDA110 and USDA123

From the 42 cultivated soybean accessions that showed a significant difference in NN when inoculated with USDA110 and USDA123 in single inoculation trials, 30 accessions with higher NN of USDA110 than USDA123 plus five accessions with higher NN of USDA123 than USDA110 were selected to be inoculated with a blend of USDA110 and USDA123 (Fig. 1; Table S2).

**Fig. 2:**
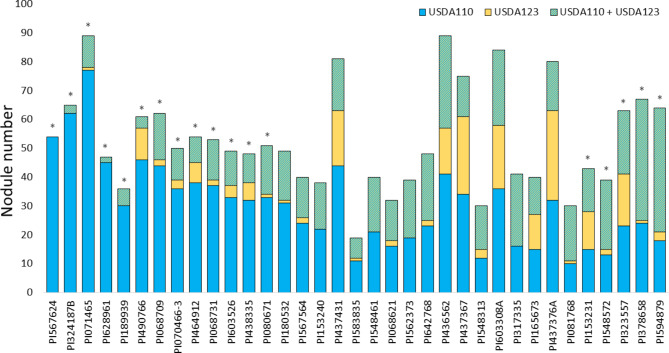
Number of nodules containing *Bradyrhizobium japonicum* strains USDA110, USDA123, or both USDA110 and USDA123 in 35 soybean accessions inoculated with mixed USDA110 and USDA123 inoculum. The asterisk (*) indicates significant difference in the number of nodules containing USDA110 vs. nodules containing USDA123 and both USDA110 and USDA123 at the 5% probability level

Strain USDA110 was highly competitive against USDA123 in 13 accessions. Among them, accessions PI567624, PI324187B, PI071465, PI628961, PI189939, PI490766, PI070466-3, PI068731, PI603526, PI438335, and PI080671 had high NN of USDA110 when singly inoculated, but plant introductions PI068709 and PI464912 had high NN of USDA123. On the other hand, strain USDA123 was highly competitive against USDA110 in five accessions, two with high NN of USDA123 in the single inoculation trial (PI153231, PI323557), and three with high NN of USDA110 (PI548572, PI378658, PI594879) in the same trial (Fig. 2; Table S3).

Out of the 35 accessions, 27 formed three different types of nodules, which consisted of USDA110, USDA123, and mixed nodules (USDA110 + USDA123 nodules). The occupancy preference of nodules in 16 of these accessions was USDA110 > mixed nodules > USDA123; seven genotypes had two types of nodules: USDA110 and mixed nodules, with a preference for USDA110, and one accession had all nodules occupied by strain USDA110 (Fig. 2). Among the accessions, the incidence of double-strain occupancy in a nodule varied from 0 to 67%, with an average of 32.9%.

### Genetic complexity controlling nodule number in soybean

**Fig. 3:**
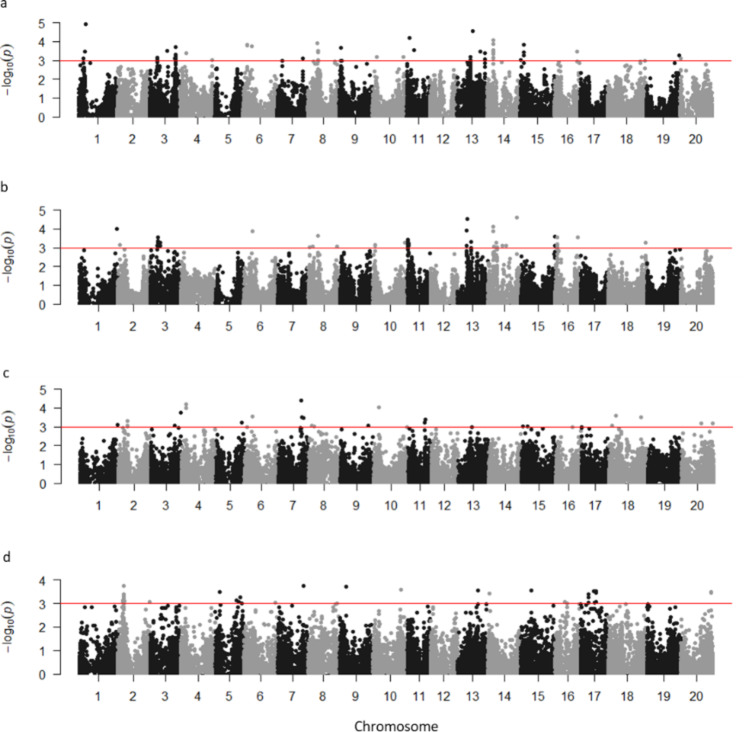
Manhattan plots of loci association with nodule number (NN) evaluated in cultivated and wild soybean accession **a** NNmax110, **b** NNmax123, **c** NNsoja110, and **d** NNsoja123. Negative log_10_-transformed *P-*values of SNPs from a genome-wide scan for NN using mixed linear model (MLM) in NNmax110 and NNmax123, and compressed mixed linear model (cMLM) in NNsoja110 and NNsoja123 that included both kinship and population structure was plotted against positions on each of the 20 chromosomes. The significant trait-associated SNPs (*P* < 0.001) were above the red threshold line

Based on the best K-value of 3 for both *G. max* and *G. soja* populations, the best fitting model controlling genomic inflation was cMLM in *G. soja* and MLM in *G. max* (Figures S1 to S4). Furthermore, genome-wide association studies (GWAS) identified a total of 50 SNPs significantly associated with NN (-Log10(*p*) > 3) when *G. max* accessions were single inoculated with USDA110 (NNmax110), 50 SNPs when the same set was inoculated with USDA123 (NNmax123), and 32 and 51 in *G. soja* single inoculated with USDA110 (NNsoja110) and USDA123 (NNsoja123), respectively (Fig. 3).

In NNmax110, 9 of the 50 significantly associated SNPs were positioned on chromosome 14 in the linkage-disequilibrium (LD) haploblock Gm14_Hap123 in the euchromatic region spanning 0.43 Mb (from 9 to 9.5 Mb). In NNmax123, 11 of the 50 SNPs were identified in the euchromatic region on chromosome 11, spanning 1.4 Mb (from 0.1 to 1.5 Mb) and positioned in five different haploblocks with one to four SNPs in each block. Only four SNPs were in common between NNmax110 and NNmax123, i.e., SNPs ss715584397 at position 11.5 Mb on chromosome 3, haploblock Gm03_Hap5; ss715593108 at position 13.8 Mb on chromosome 6; ss715620123 at 9.4 Mb on chromosome 14, haploblock Gm14_Hap123; and ss715624623 at position 33.7 Mb on chromosome 16.

In NNsoja110, six over 32 significant SNPs were located in the heterochromatic region on chromosome 11. Five of the six SNPs were located in haploblock Gs11_Hap28 (25.116–25.123 Mb), plus one SNP located 0.9 Mb downstream. For NNsoja123, 21 over 51 SNPs were found on chromosome 17, six in haploblock Gs17_Hap5 spanning 4.2 Mb (22.7–23.7 Mb) in the heterochromatic region, and 15 SNPs were located in blocks with one to six significant SNPs, or not in haploblocks. No significant SNPs in common were found between treatments NNsoja110 and NNsoja123 or interspecies.

In the final count of significant loci for NN, we kept the significant leading SNP to represent each haploblock, as well as the SNPs outside the haploblocks. As a result, 35, 35, 23, and 34 loci were detected from NNmax110, NNmax123, NNsoja110, and NNsoja123, respectively Table S4), including the four common regions between NNmax110 and NNmax123. However, each locus could only explain 0.8 to 1.5% of phenotypic variation.

## Discussion

**Fig. 4:**
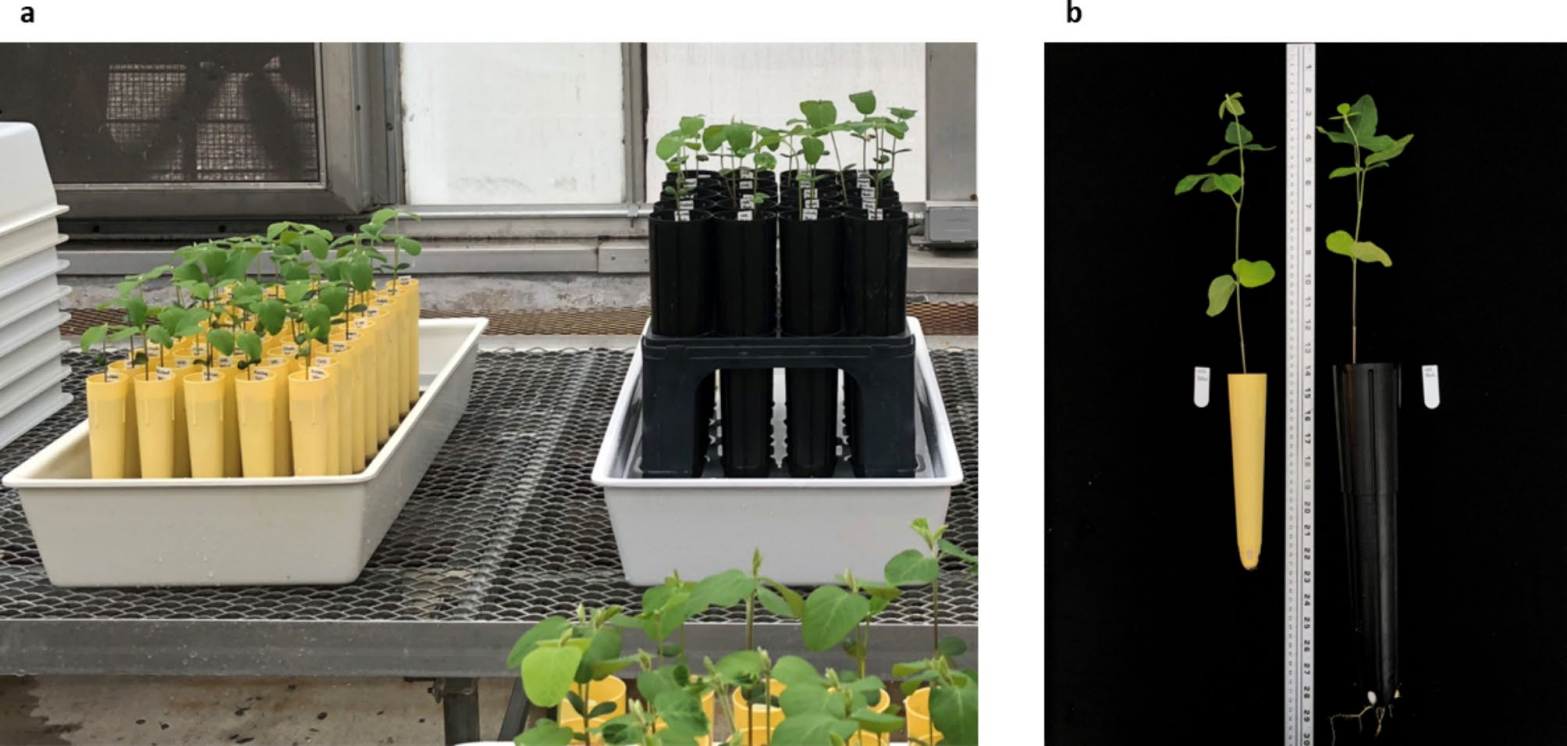
**a** Comparison of the area entailed with Cone-tainers (yellow) and Deepots (black), and **b** comparison of the development of cultivated soybean in Cone-tainers (yellow) and Deepots (black) for high-throughput phenotyping of root nodule number

Conventionally, scientists have used Leonard Jars as a growth system for the study of nodulation in legumes [35–40]. Later, some publications reported the use of Deepots for experiments with soybean cyst nematodes (Stuewe & Sons, Inc., Tangent, Oregon, USA) [41, 42]. Leonard Jars and Deepots are similar growth systems whose main advantages are controled growth, contamination control, self-irrigation, and obtaining the whole intact root for phenotyping. The area occupied by both systems is similar, approximately 12 plants/m^2^. For experiments with a large number of samples, such as in this study, establishing an efficient system of phenotyping that maximizes space is fundamental to maintaining similar growth conditions. Therefore, Cone-tainers of 164 ml (3.8 cm diameter x 21 cm depth) (Stuewe & Sons, Inc., Tangent, Oregon, USA) with the same advantages but smaller than the Deepots and Leonard Jars were used to evaluate soybean nodules. Cone-tainers were distributed in sets of 45 units/tray entailing an area of approximately 22 plants/m^2^, which increased the area by 83% compared with the Deepots and Leonard Jar systems (Fig. 4a). Moreover, soybean accession germination and growth were uniform (Fig. 4b). Nodulation depends on several environmental conditions, especially light, temperature, and nitrate concentration; this study followed the standard protocol for phenotyping nodule number in substrates without nitrate from previous reports [24, 43–45]. However, further competition studies under field conditions or in substrate with nitrate in greenhouse are essential. Although similar but not identical systems were previously reported in nodulation experiments in clover [46], pea [47], and *Astragalus* (Fabaceae) [48], this is the first report to use this system in soybean.

Likewise, developing methods to determine nodule occupancy in a large number of accessions was critical for this nodulation competition study. The rapid DNA extraction and high-throughput Kompetitive Allele Specific (KASP) genotyping method established in this study allowed us to accuraterly differentiate the types of nodules, which was verified by Sanger sequencing of nodules and USDA110 and USDA123 strain controls. In initial experiments, isolation of DNA was performed with PrepMan Ultra (Applied Biosystems); however, DNA amplification with the KASP protocol did not amplify as expected, affecting allelic discrimination, probably due to interaction between the nodule’s polysaccharides and PCR chemical reagents. It is well known that polysaccharides could inhibit PCR [49, 50].

Identifying accessions with preference for nodulation with one strain over the other was the first step in selecting genotypes of interest for the strain competition experiment. In this study, 11 of 30 accessions selected in the first screening of single inoculation with a high NN of USDA110 versus USDA123 showed the same significant preference for USDA110 nodulation compared to USDA123 in competition experiments, while two of five accessions initially selected with a high NN of USDA123 versus USDA110, had the same preference in the competition experiment as single inoculation, and the remaining three accessions preferred USDA110 to USDA123, suggesting the complexity of the strain competition mechanisms. In the competition study, the incidence of mixed nodules ranged from 0 to 67% under controlled conditions, a wide variation among the genotypes. Nodule mixed infections in soybean, and other legumes species are common and have been previously reported [24, 25, 51–55].

In the early days, Lindemann et al. [56] found that up to 32% of the nodules of soybean grown in sterilized sand contained mixed nodules when plants were inoculated with two strains. Similar results were described by Moawad and Schmidt [57], with 12–32% incidence of double occupancy, Okogun and Sanginga [58] with 34%, and Payakapong et al. [44] with 4–69% of mixed nodules. Lohrke et al. [25] reported more than one strain infected 0 to 44% of soybean nodules. Furthermore, a single nodule may contain more than one genotype of bacteroid sumbiont and may also harbor non-rhizobial species as well [51, 53, 54].

With the intent to find genotypes with restricted nodulation to the strain USDA123, Cregan and Keyser [26] screened accessions that adapted to Southern U.S. maturity groups. They counted 30 nodules per plant in cultivar ‘Lee’ and on the selected restricted 22 genotypes, 1 to 8. In the current experiment, under similar conditions, 51 nodules per plant were found in cultivar ‘Lee,’ and on three concurrent accessions PI371607, PI548461, and PI325779, the NN per plant was 35.0, 25.8, and 26.7, respectively, confirming that the NN on these accessions is smaller than cultivar ‘Lee’ when inoculated with USDA123. These authors also found that 13 genotypes demonstrated restricted nodulation with USDA123 compared to five standard cultivars; PI371607 was sowed along with cultivar ‘Williams’ in soil free of *B. japonicum* and inoculated with USDA123 and either USDA110, USDA122, or USDA138. In each competition treatment, greater than 75% of the nodules of ‘Williams’ were occupied by USDA123, whereas in all cases, less than 10% of the nodules of the PI371607 genotypes contained USDA123. The accessions genotypes excluded strain USDA123 in favor of the inoculant strain. In the present experiment, PI371607 did not significantly differ when inoculated with USDA110 (35 nodules/plant) or USDA123 (37.3 nodules/plant). Consequently, it was not included in further competition experiments in this study. Nevertheless, when PI548461 (‘Improved Pelican’), classified with restricted nodulation to USDA123 by Cregan and Keyser [26] was tested, a significantly higher number of USDA110 (52.5) nodules than USDA123 (25.8) (*P* < 0.05) was found.

Under a different approach, Cregan et al. [24] identified a genotype that restricted nodulation with USDA110, MN1-1c, and USDA138. Therefore, accession PI417566 was single inoculated with strain USDA123 and USDA110, and the NN per plant was 31 and 5, respectively. Subsequently, Lohrke et al. [25], in an equivalent experiment, tested strain USDA123 and USDA110 in the same accession and found similar results, 32 (USDA123) and 3.3 (USDA110) nodules per plant. In the present experiment, 54 and 32 nodules per plant were found in PI417566 when single-inoculated with USDA123 and USDA110, respectively, yet no statistical difference was detected.

Identifying accessions with potential nodulation restriction for USDA123 strain and specificity for superior rhizobium strain would facilitate the development of soybean cultivars with improved symbiosis through hybridization and introgression of genes from the accessions. Several USDA123-restricting and USDA110-preferring accessions were identified from this study could be a valuable source for further genetic research on this trait and soybean improvement.

Although several articles have reported in soybean restricted nodulation to specific strains of *Bradyrhizobium* or *Esinfer*, efforts to find genes that limit USDA123 nodulation have not yet been reported [24–26, 59].

In the present study, when accessions of *G. max* and *G. soja* were single-inoculated with *B. japonicum* strains USDA110 and USDA123, 35 loci were identified in treatment NNmax110 and the same number of loci in NNmax123. Moreover, 23 loci were identified in NNsoja110 and 34 in NNsoja123. From the total of 127 loci, 107 unique significant loci were identified, and 18 have been previously reported for NN or nodule-related traits in SoyBase (http://soybase.org). The significant SNP ss715590625 in NNsoja123 located at 31.4 Mb on chromosome 5 was adjacent to the gene *Glyma.05G121600* (*GmVTL1a/nodulin-21*), which is a vacuolar iron transporter-like gene indispensable for symbiotic nitrogen-fixation in nodules, annotated as a transporter of ferrous iron from the infected root cell cytosol to the symbiosome [60–62].

The region on chromosome 6 where two loci were found, ss715595435 (6,581,862 bp) in NNmax110 and the common SNP ss715593108 (13,759,357 bp) in NNmax110 and NNmax123, have been previously described by Yang et al. [63] in the interval position at 6,529,355 to 16,221,141 bp (position according to Wm82.a.2.v1, Song et al. [64], which was similar to the region described by Hwang et al. [65] for NN (12,336,655 to 14,225,129 bp). On another hand, on the same chromosome 6, Nicolás et al. [66] found a QTL in the region 44,884,659 to 49,271,843 bp, where the SNP ss715594793 (47,869,389 bp) was significant for NNsoja123.

On chromosome 10 SNPs ss715607620 in haploblock Gm10_Hap93 (43,688,879 to 46,747,178 bp) in NNmax110 and ss715607699 at 47,626,066 bp in NNmax123, were found in a similar region (43,509,883 to 48,424,003 bp) previously reported by Yang et al. [63] for six QTLs nodule-related traits.

On chromosome 11, Yang et al. [63] and Santos et al. [67] each found a significant region (intervals 2,718,971 to 6,217,092 bp and 4,234,240 to 9,107,006 bp, respectively) for NN where SNP ss715610750 (4,611,787 bp) was significant in NNmax110. Furthermore, Nicolás et al. [66] found two QTLs for NN and nodule dry weight per plant in an extensive region on chromosome 14, from 16,249,052 to 45,041,580 bp, where three significant SNPs in NNmax123 were found, ss715617892 at 22,010,302 bp, ss715618045 in haploblock Gm14_hap29 (26,079,931 to 26,911,748 bp), and ss715618900 at 43,535,411 bp. On chromosome 15 the two regions from 4,434,024 to 5,540,983 bp and from 7,537,816 to 43,037,732 bp have been described for nodule size by Hwang et al. [68] and for NN by Santos et al. [67], respectively, where three significant SNPs were found, ss715622828 (5,241,013 bp) in haploblock Gm15_Hap51 (5,218,552 to 5,252,046 bp) in NNmax110, ss715620209 (9,445,842 bp) in NNsoja110, and SNP ss715621072 (16,434,045 bp) in NNsoja123.

SNP ss715624623 on chromosome 16 was among the four significantly associated common markers in NNmax110 and NNmax123, where seven putative genes reside. Three of them, *Glyma.16G175600* (*GmUGT1*), *Glyma.16G175900* (*GmUGT7*), and *Glyma.16G176000* (*GmIF7GT5*), are related to symbiosis and are involved in the isoflavonoid pathway coding for isoflavone 7-O-glucosyltransferase, which plays important roles in plant-microbe interaction and is essential for establishing symbiosis with *B. japonicum* [69]. Moreover, isoflavonoids genistein and daidzein are also the primary inducers of *nod* gene expression in *B. japonicum* [70–72], however no significant terms were found after the GO analysis.

On chromosome 19, the SNP ss715635907 at 49,239,109 bp was significant in NNmax110, and the region from 49,210,133 to 49,243,536 was also described by Nicolás et al. (2006) for nodule dry weight. On chromosome 20, two of the significant SNPs found in NNsoja123, ss715638590 (44,971,784 bp) in haploblock Gs20_Hap55 (44,971,784 to 44,992,638 bp), and ss715638603 (45,139,632 bp), were previously described by Hwang et al. [68], and one significant SNP in NNsoja110, ss715637162 (29,015,399 bp) in the extended region (2,717,137 to 34,051,592 bp) was described by Santos et al. [67] for root nodule dry weight. Moreover, when four treatments were compared, four significant loci overlapped treatments NNmax110 and NNmax123, i.e., SNPs ss715584397 at position 11.6 Mb on chromosome 3, haploblock Gm03_Hap5; ss715593108 at position 13.8 Mb on chromosome 6; ss715620123 at 9.4 Mb on chromosome 14, haploblock Gm14_Hap123; and ss715624623 at position 33.7 Mb on chromosome 16.

## Conclusions

The detection of multiple QTLs associated with NN, the small number of common or overlapped QTLs between inoculation treatments, and the small effects at each locus implied the complexity of the trait and complex signal exchange between rhizobia and the host to establish symbiosis. This study also supported the evidence that host-controlled restriction or nodulation preference was strain dependent.

## Methods

### Selection of diverse sets of soybean accessions for evaluation

A total of 18,480 *G. max* and 1168 *G. soja* accessions were provided by the USDA Soybean Germplasm Collection located at Urbana-Champaign, Illinois, USA (https://www.ars-grin.gov/) and previously genotyped with the SoySNP50K assay, containing more than 50,000 SNPs [28]. Analysis of the dataset revealed that 4,303 *G. max* (23%) and 362 *G. soja* (30%) accessions were at least 99.9% identical to another accession in the Collection. From the remaining 14,186 cultivated and 806 wild soybean accessions, diverse sets of 1,417 cultivated and 81 wild soybean accessions were selected using the following procedures: pairwise distances among 14,186 cultivated and among 806 wild soybean accessions were estimated by calculating the ratio of the number of SNPs with different alleles to the total number of loci between paired accessions. The cultivated and wild soybeans were clustered into 1,418 and 82 clusters, respectively, equal to approximately 10% of the cultivated and wild soybean accessions in the Collection. From each cluster, one accession with the largest average distance to other accessions was chosen. Further analysis showed that the two sets could capture > 90% of the genetic diversity in cultivated and wild soybean collections based on the ratio of the number of polymorphic SNPs in the selected accessions versus the total number of polymorphic SNPs among all cultivated and wild soybean accessions. However, due to the lack or deficiency of seeds for some accessions, only 1,321 accessions of the 1,418 *G. max* (landrace and modern cultivars) and 69 accessions of the 81 *G. soja* were used in this study (Table S1). These accessions represented 51 countries and all maturity groups (000 to X).

### Evaluation of nodule number in soybean after single inoculation with USDA110 and USDA123

Plants were grown in a greenhouse located in the Beltsville Agricultural Research Center, USDA, Maryland, USA. Conditions were the same for all the experiments, natural sunlight was supplemented by incandescent light to a photoperiod of 16 h and a temperature range of 25 ± 7 °C. Cone-tainers of 164 ml (3.8 cm diameter x 21 cm depth) (Stuewe & Sons, Inc., Tangent, Oregon, USA) were used to grow plants. A medium cotton ball was placed on the bottom of each Cone-tainer and filled with prewashed sand. Cone-tainers were supported by seedling flats of 162 cells (9 × 18), distributed in sets of 45 units leaving an empty cell between each Cone-tainer (5 columns x 9 rows). Seedling flats and Cone-tainers were placed in trays filled with 3 inches of tap water. Sand and cotton balls were autoclaved, and Cone-tainers, flats, and trays were disinfected for 5 min in a 1:4 bleach solution, then rinsed three times with water. For disinfection and sowing, a seedling flat of 162 cells was modified into a compartmentalized strainer. First, the size was reduced to 45 cells (5 × 9 cells), and on the bottom of each cell, a plastic mesh # 10 was glued. Next, seeds and plastic labels of 45 accessions were distributed over the seed strainer, then disinfection took place by sequentially immersing the seed strainer, first in the tray with 70% ethanol for 30 s, then rinsed in water, washed in 1:4 bleach solution for 4 min in another tray, and finally, rinsed four times with water by transferring the seed strainer tray by tray. The seed strainer had the same distribution as the experimental unit of the Cone-tainers. Therefore, four seed strainers were used at a time per disinfection. With this method, seeds of 180 accessions were disinfected in 6 min.

The experiment was conducted in a randomized complete block design with 1,321 *G. max* and 69 *G. soja* accessions in four replicates. For each replicate, four seeds were sowed per Cone-tainer, and after four days, seedlings were thinned to one and wetted with Nitrogen-free Hoagland-Arnon plant nutrient solution [73]. Five-day-old plants were single-inoculated with 2 ml of suspension (10^8^ CFU/ml^-1^) of *B. japonicum* USDA110 and USDA123 strains, respectively. Water in trays was changed weekly. After 30 days, roots were harvested, washed, dried, and frozen, and then nodules were removed and counted. Thirty-six un-inoculated ‘Williams 82’ plants were included as controls distributed in four units along the benches, and no nodules were found on them when phenotyped.

### Evaluation of nodule number and determination of nodule occupancy in soybean after double inoculation with USDA110 and USDA123

A completely randomized design experiment with four replicates was performed on 35 accessions that showed a significant difference in NN after single inoculation with USDA110 and USDA123, respectively. Five-day-old plants were double inoculated with a 2 ml blend suspension of equal concentration (10^8^ CFU/ml^− 1^) and volume (1:1) of USDA110 and USDA123. After 30 days, roots were harvested, washed, and disinfected with alcohol 75%, dried up for 20 min at 28 °C, then frozen for further analysis. After that, nodules were detached from roots and placed in a sterile 96-well plate (0.8 ml), one nodule per well, then lyophilized overnight for DNA extraction. A single steel bead of 2.4 mm was placed in each well, and the plate was sealed with thermo-aluminum film. Nodules were ground at 30 Hz for 30 s in a Retsch MM400 mixer mill. Plates were centrifuged at 5,200 rpm for 5 min, and 500 µl of distilled water was added to each sample. Plates were sealed, vortexed, spun down and sat for 20 min. From the supernatant, 100 µl was transferred to a new 96-well PCR plate, sealed, and centrifuged at 5,200 rpm for 10 min; the supernatant was removed without disturbing the pellet, then 50 µl of distilled water was added to the pellet, the plate was briefly vortexed, then heated at 100 °C for 10 min, centrifuged at 3,000 rpm for 5 min, and finally, 20 µl of the supernatant was taken and transferred into a new 96-well plate. DNA was diluted to 10% for the PCR reaction.

To determine nodule occupancy of USDA110 and USDA123 in soybean after double inoculation of USDA110 and USDA123, a set of 23 Kompetitive Allele Specific PCR (KASP) markers were designed using WASP Web-based Allele Specific Primer Design Tool (https://bioinfo.biotec.or.th/WASP) [74], based on the genome sequence of USDA110 (https://www.ncbi.nlm.nih.gov) and USDA123 (http://genome.jgipsf.org). Among these, two markers, rhi_snp2 (Allele-1 forward primer sequence: GAAGGTGACCAAGTTCATGCTCCAGGATGATGATCACGCCC, Allele-2 forward primer sequence: GAAGGTCGGAGTCAACGGATTCCAGGATGATGATCACGCCG, and reverse primer sequence: GCACAGGTCAGCTCCTACTG), and rhi_snp24 (Allele-1 forward primer sequence: GAAGGTGACCAAGTTCATGCTGTGGTCTCGTTGCTGGCT, Allele-2 forward primer sequence: GAAGGTCGGAGTCAACGGATTGTGGTCTCGTTGCTGGCC, and reverse primer sequence GACAACATGATCCTCGCGCTC), had the best amplification and allelic discrimination. Thus, both markers were chosen to test nodule occupancy on 35 *G. max* accessions. Nodules were genotyped by KASP markers in a final reaction volume of 5 µL with 2.5 µL of 2x KASP MasterMix Low Rox 5000 V4.0 TF (LGC Genomics), 0.07 µl of the primers mix containing 12 µM of each forward primer plus 30 µM of the reverse primer, and 5–20 ng of genomic DNA. QuantStudio 6 Flex (Applied Biosystems, Thermo Fisher Scientific) was used for PCR under the following cycling conditions, activation for 15 min at 94 °C; 10 cycles of 20 s at 94 °C and 60 s at 61–55 °C (annealing temperature for each cycle was reduced by 0.6 °C per cycle); 26 cycles of 20 s at 94 °C and 60 s at 55 °C, followed by 15 cycles of 20 s at 94 °C and 60 s at 57 °C. Fluorescence detection was performed at the end of the run for 30 s at 35 °C, and data were analyzed with Quant Studio Real-Time PCR software (Applied Biosystems, Thermo Fisher Scientific). For the validation of the phenotyping results, 12, 13, and 18 bacteroids from 3 mixed nodules were used for Sanger-sequencing at seven loci defined in the Bradyrhizobium Multilocus Sequence Analysis [75]. A t-test was performed to determine the difference in nodule occupancy between USDA110 and USDA123 within soybean accession.

### Variance analysis and detection of loci controlling nodule number in soybean

Analysis of variance and heritability were performed for the NN in *G. max* single inoculated with *B. japonicum* strains USDA110 (NNmax110) and USDA123 (NNmax123) and in *G. soja* single inoculated with USDA110 (NNsoja110) and USDA123 (NNsoja123). To determine the complexity of NN, the number of loci, and their effects on the trait were analyzed through genome-wide association studies (GWAS). Population structure was analyzed by ADMIXTURE v1.22 [76] with *K* from 2 to 10 for *G. max* and 2 to 5 for *G. soja*, and a 10-fold cross-validation (CV) procedure was performed with 30, 100, 500, 1000, and 2000 seeds for each *K*-value. The most likely *K*-value was determined using the CV values. Consequently, marker and NN associations were tested in Tassel [77] using the general linear model (GLM) with population structure, regular mixed linear model (MLM), and compressed mixed linear model (cMLM), considering kinship matrix and population structure. The significance threshold for SNP-trait associations was set at the empirical value of *P* < 0.001 or -Log_10_ (*P*) > 3. The haplotype blocks of the soybean genome were determined in Haploview 4.2 [78], and significant QTLs were compared with QTLs for NN and other nodule-related traits found in SoyBase (http://soybase.org) and previous reports. When a given QTL was outside the previously described interval position, we considered it as a novel QTL. Genes located in the significant regions were searched for expression abundance in nodule-related traits in Phytozome (https://phytozome-next.jgi.doe.gov/) [79], genes involved in the rhizobia-mediated nodulation were kept. Finally, a gene ontology (GO) analysis was performed for these genes in agriGO toolkit v.2.0 (http://systemsbiology.cau.edu.cn/agriGOv2) [80]. In case of less than ten candidate genes, we added some random genes to enable the program to perform the GO analysis.

### Electronic supplementary material

Below is the link to the electronic supplementary material.

Supplementary Material 1

Supplementary Material 2

Supplementary Material 3

Supplementary Material 4

Supplementary Material 5

## Data Availability

The genotypic dataset for the diverse set of wild and cultivated soybean is available at https://www.soybase.org/snps/, the phenotypic dataset of the accessions is in the Table S1.

## List of abbreviations

SNP: Single-nucleotide polymorphism
NN: Nodule number
NS: Non-significant
GWAS: Genome-wide association studies
LD: Linkage disequilibrium
QTL: Quantitative trait loci
CV: Cross-validation
GLM: General linear model
MLM: Mixed linear model
cMLM: Compressed mixed linear model
KASP: Kompetitive allele specific PCR
PCR: Polymerase chain reaction
DNA: Deoxyribonucleic acid
GO: Gene ontology
