# Supplementary material for: Genetic variation and genetic complexity of nodule occupancy in soybean inoculated with USDA110 and USDA123 rhizobium strains
Source: BMC Genomics. 2023 Sep 4;24:520. doi: 10.1186/s12864-023-09627-4 (PMC10478483; doi:10.1186/s12864-023-09627-4)
Supplement: Supplementary file 4 — Supplementary Material 4 [file 12864_2023_9627_MOESM4_ESM.pdf]

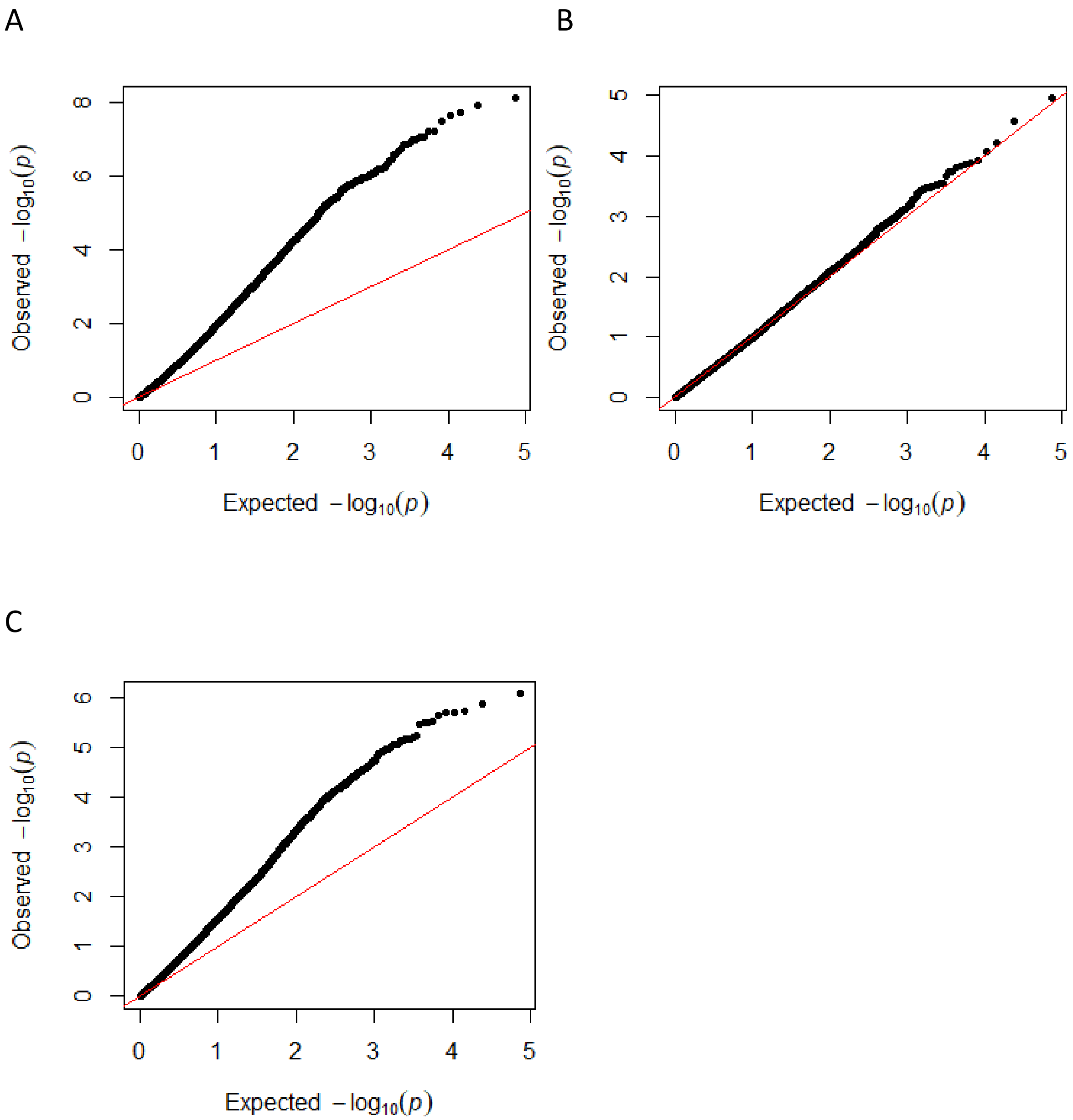

Figure S1 Quantile-quantile plot of association analysis for nodule number in *G. max* inoculated with *Rhizobium japonicum* USDA110 using **A** general linear model (GLM), **B** mixed linear model (MLM), and **B** MLM with compression (MLMc).

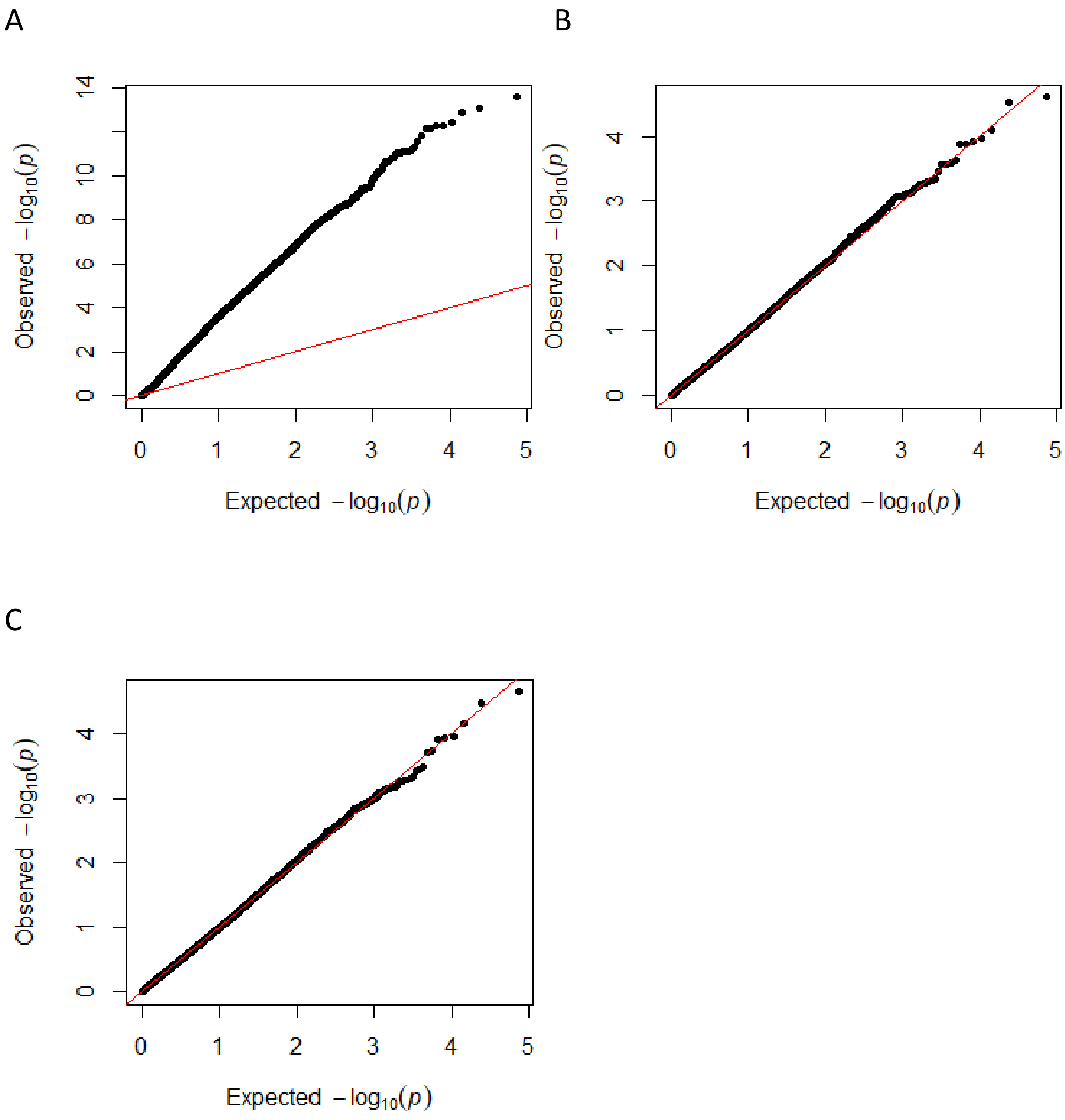

Figure S2 Quantile-quantile plot of association analysis for nodule number in *G. max* inoculated with *Rhizobium japonicum* USDA123 using **A** general linear model (GLM), **B** mixed linear model (MLM), and **B** MLM with compression (MLMc).

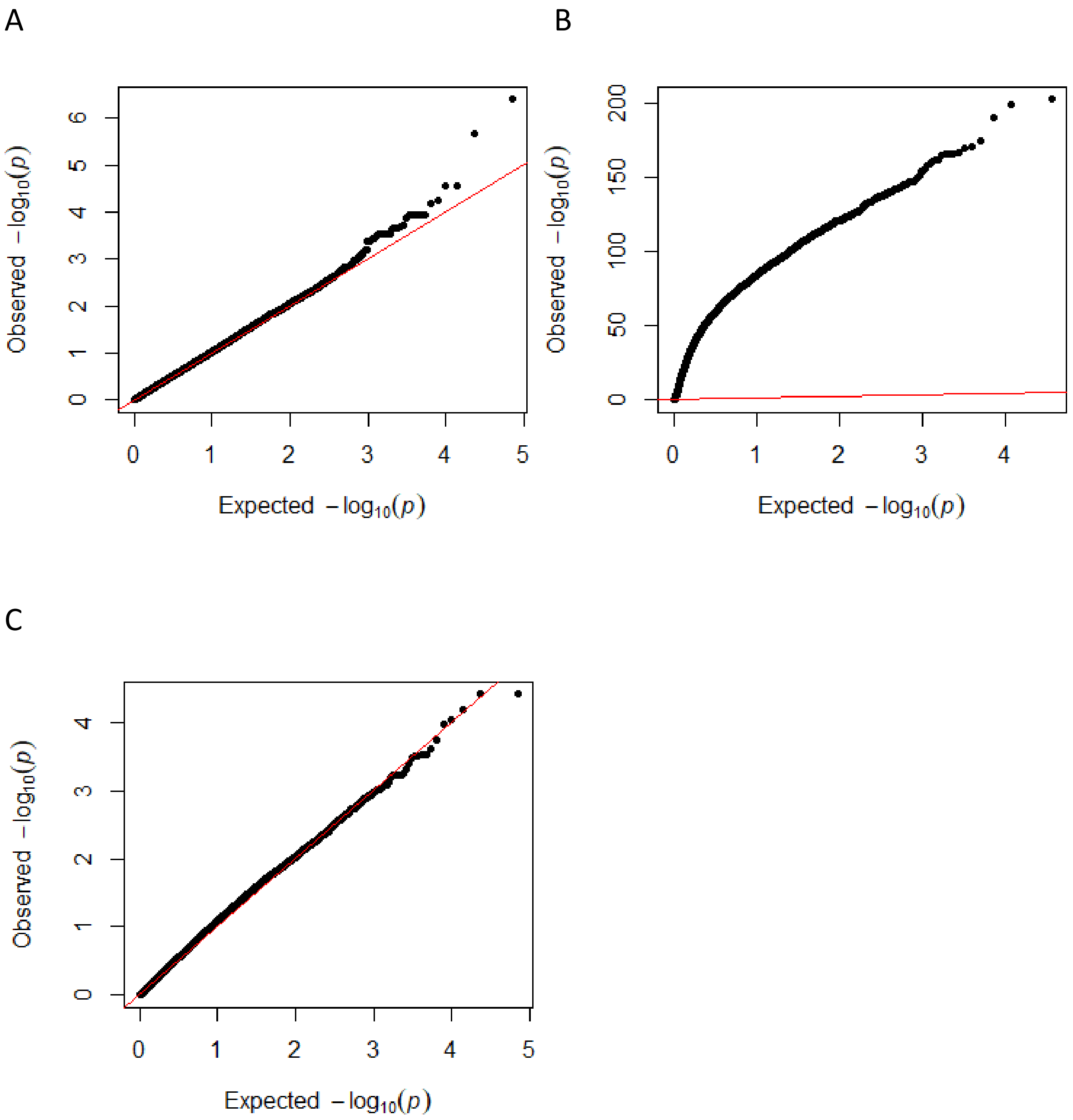

Figure S3 Quantile-quantile plot of association analysis for nodule number in *G. soja* inoculated with *Rhizobium japonicum* USDA110 using **A** general linear model (GLM), **B** mixed linear model (MLM), and **B** MLM with compression (MLMc).

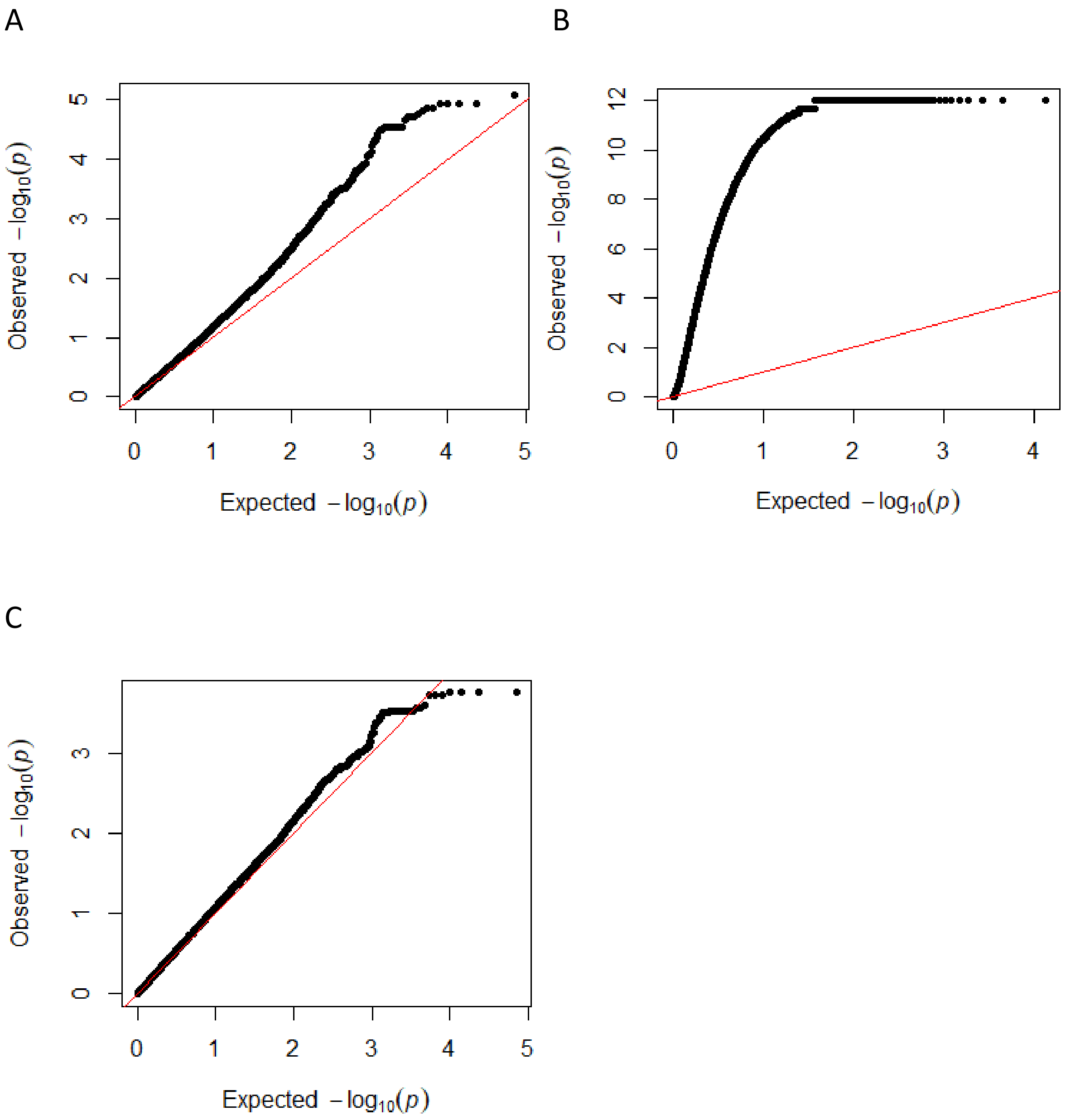

Figure S4 Quantile-quantile plot of association analysis for nodule number in *G. soja* inoculated with *Rhizobium japonicum* USDA123 using **A** general linear model (GLM), **B** mixed linear model (MLM), and **B** MLM with compression (MLMc).
